# The clinicopathological significance and prognostic value of EMMPRIN overexpression in cancers: evidence from 39 cohort studies

**DOI:** 10.18632/oncotarget.19740

**Published:** 2017-07-31

**Authors:** Hengwei Fan, Wanwan Yi, Chenxing Wang, Jisheng Wang

**Affiliations:** ^1^ Department of Hepatobiliary Surgery, The 2nd Affiliated Hospital & Yuying Children’s Hospital, Wenzhou Medical University, Zhejiang 325027, China; ^2^ Department of Nuclear Medicine, Shanghai Tenth People’s Hospital, Tongji University, Shanghai 200072, China

**Keywords:** EMMPRIN, prognosis, clinicopathological significance, cancer, meta-analysis

## Abstract

Extracellular matrix metalloproteinase inducer (EMMPRIN) has been reported to be associated with tumor formation and invasion in many studies. However, the clinicopathological significance and prognosis of EMMPRIN in cancer patients remains inconclusive. Therefore, we conducted a meta-analysis to assess the predictive potential of EMMPRIN in various cancers. By searching Pubmed, Cochrane library database and web of science comprehensively, 39studies with 5739 cases were included in our meta-analysis. The results indicated that EMMPRIN overexpression was significantly associated with poor outcome of cancers (HR=2.46, 95% CI: 2.21-2.75, P<0.0001). In addition, a significant relation was found between EMMPRIN overexpression and clinicopathological features, such as tumor stage (T3+T4/ T1+T2, OR=1.87, 95% CI:1.64-2.12, P<0.0001), tumor differentiation (poor/ well+ moderate, OR=1.09, 95% CI:1.60-2.23, P<0.0001), clinical stage (III+IV /I +II, OR=1.96, 95% CI:1.69-2.27, P<0.0001) and nodal metastasis (positive/negative, OR=2.37, 95% CI:1.93-2.90, P<0.0001). However, the expression of EMMRIN was not significantly associated with tumor stage in cervical cancer (OR=1.35, 95%CI: 0.73-2.48, P=0.33). In conclusion, EMMPRIN overxepression is significantly associated with clinicopathological characteristics and prognosis of cancers. Thus, EMMPRIN may be regarded as a promising bio-marker in predicting the clinical outcome of patients in cancers and could be used as the therapeutic target during clinical practices.

## INTRODUCTION

Cancer is a genetically and clinically diverse disease, with a tremendous amount of genetic heterogeneity across various malignant tumor types, invading and destroying nearby parts of the normal tissues [1]. The incidence and death rates of cancer are increasing in many cancer types, such as liver cancer, lung cancer and prostate cancer [2]. Besides, the survival rate of cancer patients tends to be poor for the lack of diagnostic methods with sensitivity and specificity in developing countries [3]. Latest research results predicted that biomarkers can be useful during the detection of cancers [4].

Extracellular matrix metalloproteinase inducer (EMMPRIN, basigin, HAb18G, also known as CD147) is a type I transmembrane glycoprotein of the immunoglobulin superfamily with two immunoglobulin-like domains [5, 6]. EMMPRIN has been shown to be involved in various physiological as well as pathophysiological processes such as proliferation, migration, inflammation reaction and tumor invasion [7, 8]. An increasing number of studies have demonstrated that EMMPRIN is associated with tumor growth, invasion and angiogenesis in many malignant cancer, such as breast carcinoma [9], hepatocellular carcinoma [10] and prostate cancer [11], by regulating the expression of matrix metalloproteinases (MMPs) and vascular endothelial growth factor (VEGF) [12]. MMPs have been shown to decrease the angiogenesis of tumor cells and the expression of extracellular matrix, thereby contributing to tumor progression [13]. Recently, some research data indicated that expression of EMMPRIN was obviously higher in tumor tissues than adjacent normal tissues, indicating that EMMPRIN might be useful for the prediction of prognosis in cancers.

In this study, we performed a systematically meta-analysis to investigate the relationship between EPPRIN and cancers. The aim of this study is to evaluate the clinical significance of EPPRIN and its potential value when served as a prognostic indicator.

## RESULTS

### Search results and study characteristics

**Figure 1:**
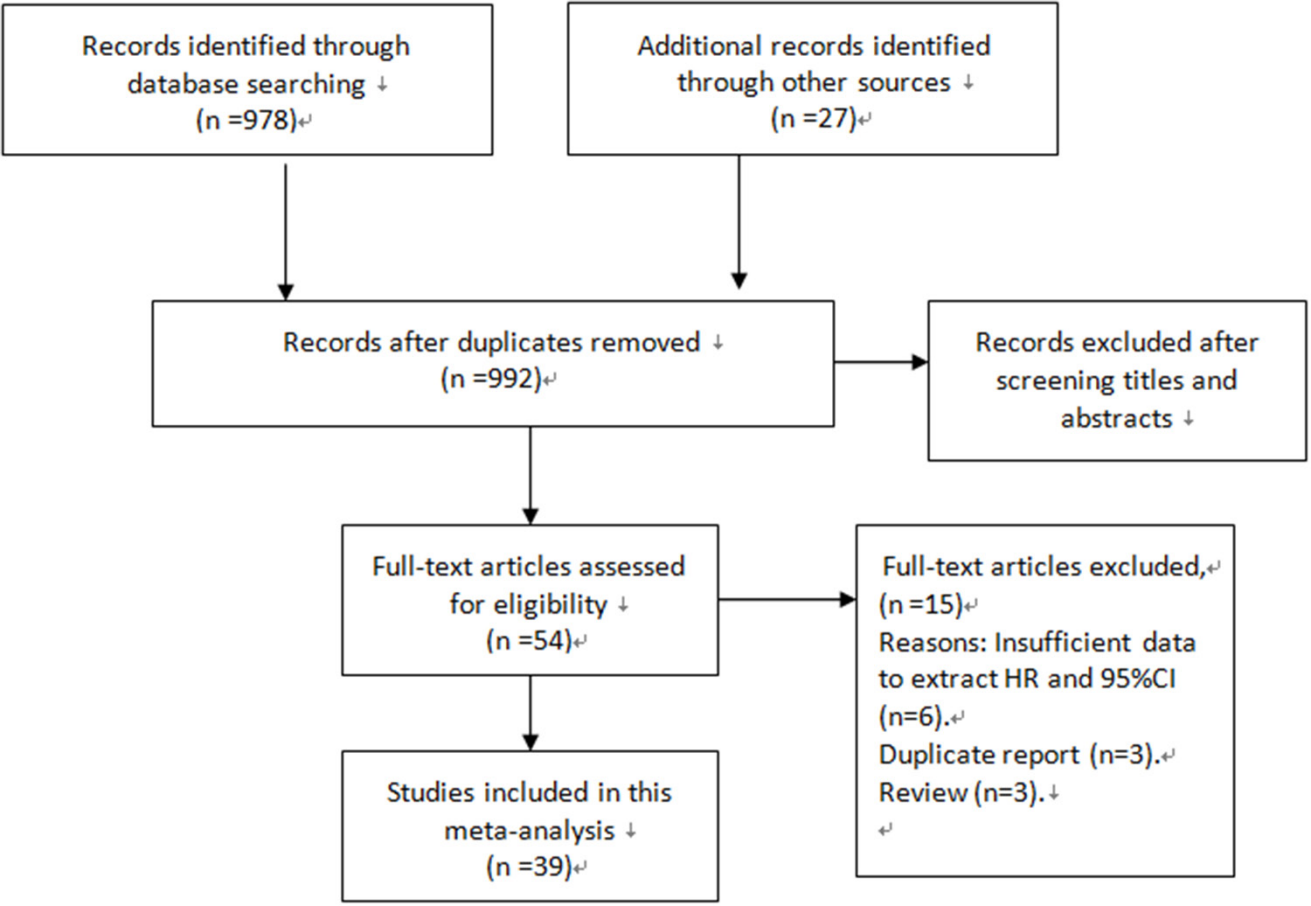
Flow diagram of the study selection process

As presented in Figure 1, 992 potentially eligible studies from the databases were retrieved after duplicates removed. Through a carefully screening process, 938 articles were excluded. Of the remaining 54 studies, 15 studies were excluded for they did not meet the inclusion criteria. Finally, 39 cohort studies were included in our meta-analysis [13–51].

The major characteristics of studies included were listed in Table 1. Among them, 29 were conducted in China, 3 from Germany, 2 from Portugal, 2 from Norway and 3 from America, Finland and Brazil respectively. We included a total of 5739 cases with different types of tumors, including bladder carcinoma, renal carcinoma, prostate carcinoma, penis carcinoma, colorectal cancer, breast cancer, thyroid carcinoma, ovarian carcinoma, glioblastoma. The testing methods of EMMPRIN were classified as immunohistochemistry (IHC) and tissue microassay. IHC staining was carried out using the paraffin-embedded block of cancer patients’ tissues compared to corresponding normal tissue, and the percentage of positive cells was calculated. The cut-off value was also list in Table 1.

**Table 1:** Characteristics of 39 pooled studies evaluating the association between EMMPRIN overexpression and cancer

First author	Year	Country	Cancer type	Sample size	Mean age	Out comes	RR (95% CI)	Testing method	Cut-off value
Zhaodong Han1	2008	China	Bladder carcinoma	58	57.2± 11.2	PFS	3.66 (1.04-12.79)	IHC	3+ (>51%)
Zhaodong Han2	2008	China	Renal carcinoma	52	56.8± 10.8	PFS	3.06 (0.82-11.44)	IHC	As above
Zhaodong Han3	2008	China	Prostate carcinoma	101	73.5± 12.3	PFS	4.87 (1.77-13.41)	IHC	As above
Zhaodong Han4	2008	China	Penis carcinoma	17	46.5± 9.2	PFS	2.38 (0.34-25.30)	IHC	As above
Zhaodong Han5	2008	China	testis carcinoma	17	48.6± 12.7	PFS	1.79 (0.22-19.94)	IHC	As above
Albrecht Stenzinger	2011	Germany	Colorectal cancer	285	67	OS	3.09 (1.91-5.02)	Tissue microassay	NM
Jung-Woo Choi	2014	China	Bladder cancer	360	69	OS	1.15 (0.50-2.67)	Tissue microassay	Scores 3
WeiDe Zhong	2010	China	Bladder cancer	101	68	PFS/OS	3.31 (1.07-15.72)	IHC	1 (>10%)
HUI TAN	2008	China	Thyroid carcinoma	156	46	PFS	3.31 (1.07-15.72)	IHC	3+ (>51%)
Xiaoyan Xua	2013	China	Non-small cell lung cancer	192	60	OS	6.63 (2.46-17.90)	IHC	3+ (>51%)
J. Afonso	2011	Portugal	Bladder carcinoma	77	71	PFS/OS	3.25 (1.02-10.39)	IHC	1 (>5%)
Xinjie Yang	2010	China	Adenoid cystic carcinoma	72	58	OS	2.78 (1.25-6.19)	IHC	NM
YauHua Yu	2015	America	squamous cell Carcinoma of the oral tongue	31	60	PFS/OS	2.82 (0.60-13.26)	IHC	Grade 2 (>25%)
Pascale Fisel	2015	Germany	Clear cell renal cell Carcinoma	186	64	OS	5.50 (2.50-12.10)	IHC	Score 3
Daniel Buergy	2009	Germany	Colorectal cancer	40	58	OS	2.50 (0.27-23.55)	IHC	>30%
Ovarian Cancer	2007	Finland	Ovarian cancer	282	61	PFS	1.32 (0.98-1.80)	IHC	>10%
Ben Davidson	2003	Norway	Ovarian carcinoma	41	59	OS	2.10 (0.76-5.81)	IHC	NM
Jian Gu	2008	China	Pediatric gliomas	45	62	PFS	0.32 (0.11-2.09)	IHC	>51%
Songlin Piao	2012	China	Salivary duct carcinoma	35	59	PFS/OS	2.95 (1.25-6.94)	IHC	Score 6
Fangfang Liu	2010	China	Breast carcinoma	110	53	PFS/OS	2.18 (0.61-7.81)	IHC	>30%
Antônio Talvane	2012	Brazil	Gastrointestinal stromal tumors	64	62	OS	1.13 (0.24-5.25)	IHC	Score 3
Min Yang	2013	China	Glioblastoma	206	57	OS	2.42 (1.35-4.18)	IHC	Score 3
Wei Wu	2008	China	Gallbladder carcinoma	60	52	OS	0.49 (0.21-1.72)	IHC	>75%
Tiefu Chen	2010	China	Primary cutaneous Malignant melanoma	150	53	PFS/OS	7.32 (1.19-20.29)	IHC	>10%
YiJun Xue	2011	China	Bladder cancer	118	58	OS	2.33 (1.15-4.73)	IHC	>51%
Ying Liu	2013	China	Basal-like breast cancer	126	56	PFS/OS	5.41 (0.74-39.49)	IHC	NM
Shaojun Zhu1	2013	China	Colorectal cancer	163	53	OS	8.88 (5.52-14.82)	IHC	Score 3
Shaojun Zhu2	2013	China	Colorectal cancer	194	53	OS	3.51 (2.03-6.08)	IHC	As above
Shaojun Zhu3	2013	China	Colorectal cancer	213	53	OS	1.89 (1.06-3.38)	IHC	As above
Zhaodong Han1	2009	China	Prostate Cancer	39	74	OS	4.49 (0.29-69.18)	IHC	Score 2 (>25%)
Zhaodong Han2	2009	China	Prostate Cancer	34	74	OS	3.54 (0.24-51.94)	IHC	As above
Che Zhang	2010	China	Intrahepatic Cholangiocarcinoma	49	66	OS	0.98 (0.76-2.01)	IHC	>51%
Tongwei Chu	2011	China	Pediatric Medulloblastoma	55	59	OS	3.50 (1.60-5.10)	IHC	Grade 2 (>10%)
Xiaoxia Gou	2014	China	Laryngeal	48	64	OS	4.87 (0.47-23.50)	IHC	Score 3
Xinwen Zhong	2013	China	Pulmonary Adenocarcinoma	180	68	OS	2.01 (1.26-3.21)	IHC	Score 3 (>51%)
K Boye	2012	Norway	Colorectal cancer	277	NR	OS	3.30 (1.40-7.80)	IHC	Score 2 (>25%)
Luís SilvaMonteiro	2014	Portugal	Oral Squamous Cell Carcinomas	74	62	OS	3.89 (1.11-13.71)	IHC	Score 5
XingZhu Ju	2008	China	Cervical Cancer	82	52	PFS	1.23 (0.52-2.90)	IHC	>51%
XinQiong Huang	2014	China	Cervical Cancer	132	51	PFS	5.12 (2.56-12.78)	IHC	>25%
LingMin Kong	2011	China	Hepatocellular carcinoma	54	60	OS	2.13 (0.78-5.79)	Tissue microassay	Score 3
Shu Zhao	2013	China	Ttriple-negative breast cancer	127	47	OS	2.68 (1.08-6.66)	IHC	NM
Li Tian1	2013	China	Astrocytic glioma	182	65	OS	2.57 (1.41-4.83)	IHC	Score 3
Li Tian2	2014	China	Astrocytic glioma	151	65	OS	4.52 (2.88-10.96)	IHC	As above
Li Tian3	2015	China	Astrocytic glioma	125	65	OS	6.61 (3.62-13.21)	IHC	As above
Dake Chu	2013	China	Gastric cancer	223	60	PFS/OS	1.59 (1.05-2.40)	IHC	Score 3
Weide Zhong	2012	China	Prostate cancer	240	62	OS	3.08 (1.62-5.85)	Tissue microassay	NM
Shaojun Zhu	2015	China	Hepatocellular carcinoma	50	65	PFS	2.41 (1.61-13.70)	IHC	>25%
Quan Zhou	2011	China	Osteosarcoma	65	55	PFS/OS	5.33 (0.57-49.56)	IHC	>51%

NR: not reporte; IRS: immunoreactivity score.

### EMMPRIN overexpression and survival in cancers

**Figure 2:**
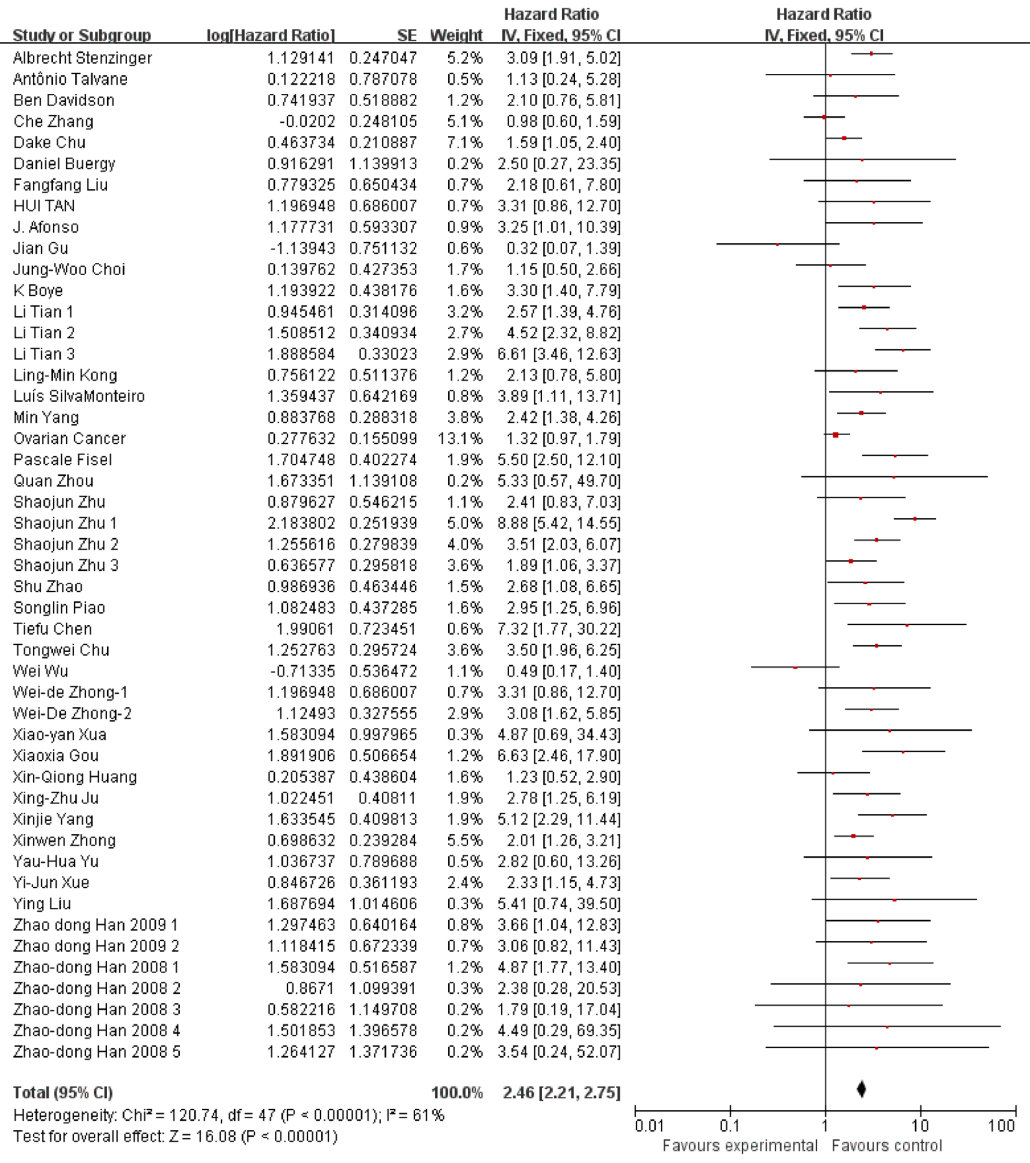
Association between EMMPRIN overexpression and the outcome of cancer patients

We used Hazard ratio (HR) and the corresponding 95% confidence interval (CI) to estimate the prognostic value of EMMPPRIN overexpression in cancers. A fixed-effect model was used to conduct the analysis due to the Heterrogeneity test (I^2^=61%, P<0.00001). The results indicated that EMMPRIN was significantly associated with OS in cancers (HR=2.46, 95% CI: 2.21-2.75, P<0.0001) (Figure 2).

**Figure 3:**
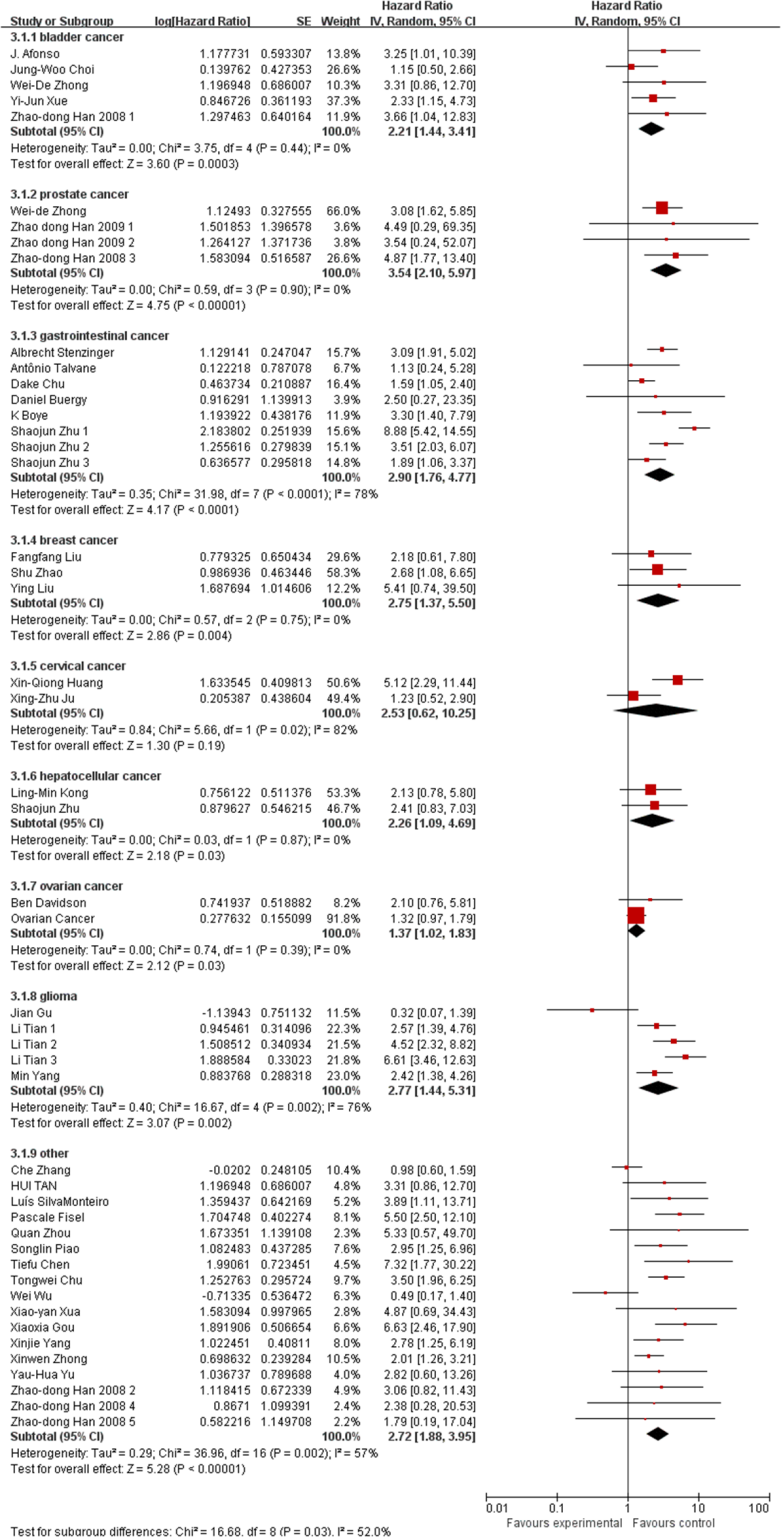
Subgroup analysis results based on tumor type

**Figure 4:**
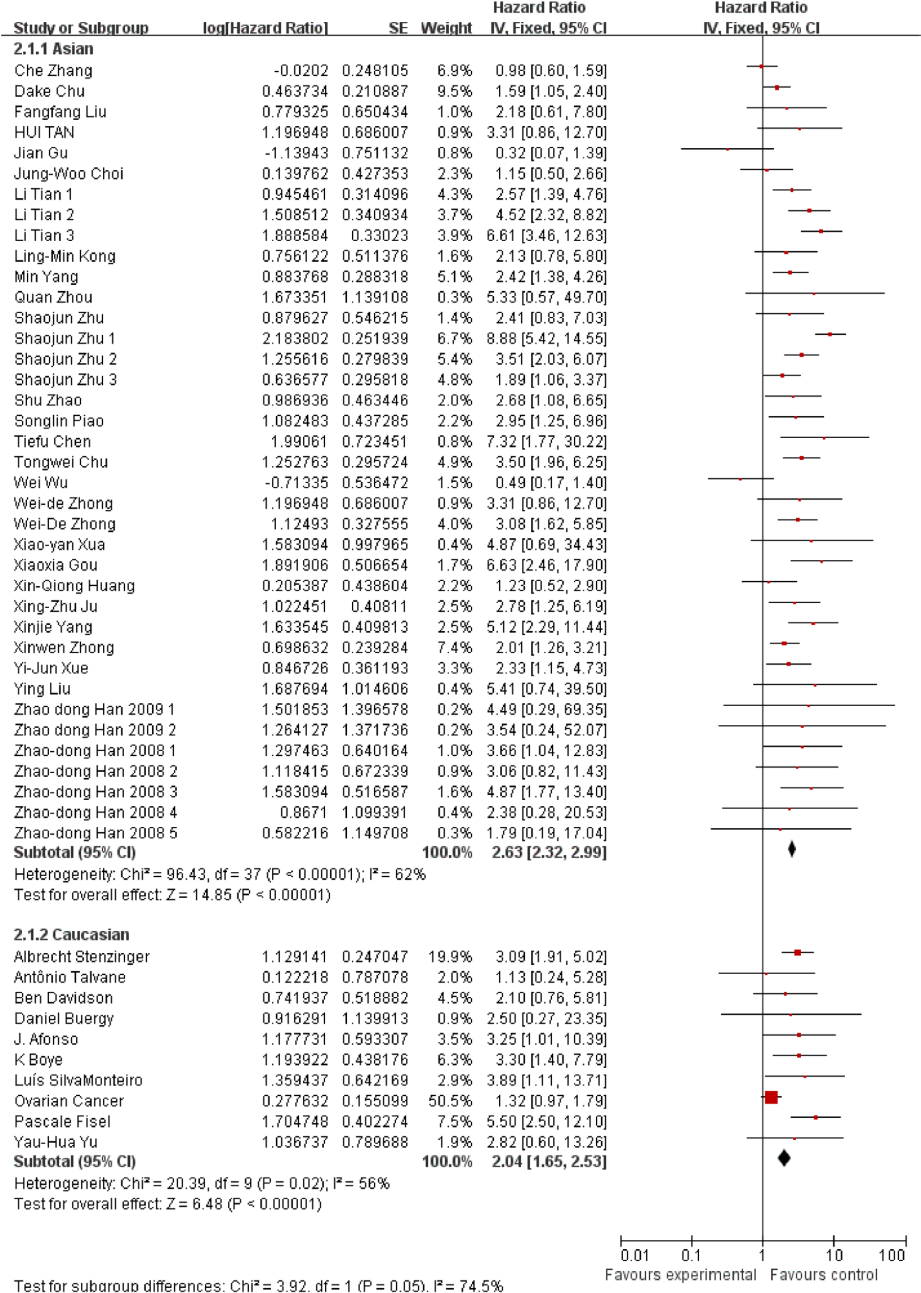
Subgroup analysis results based on ethnicity

**Figure 5:**
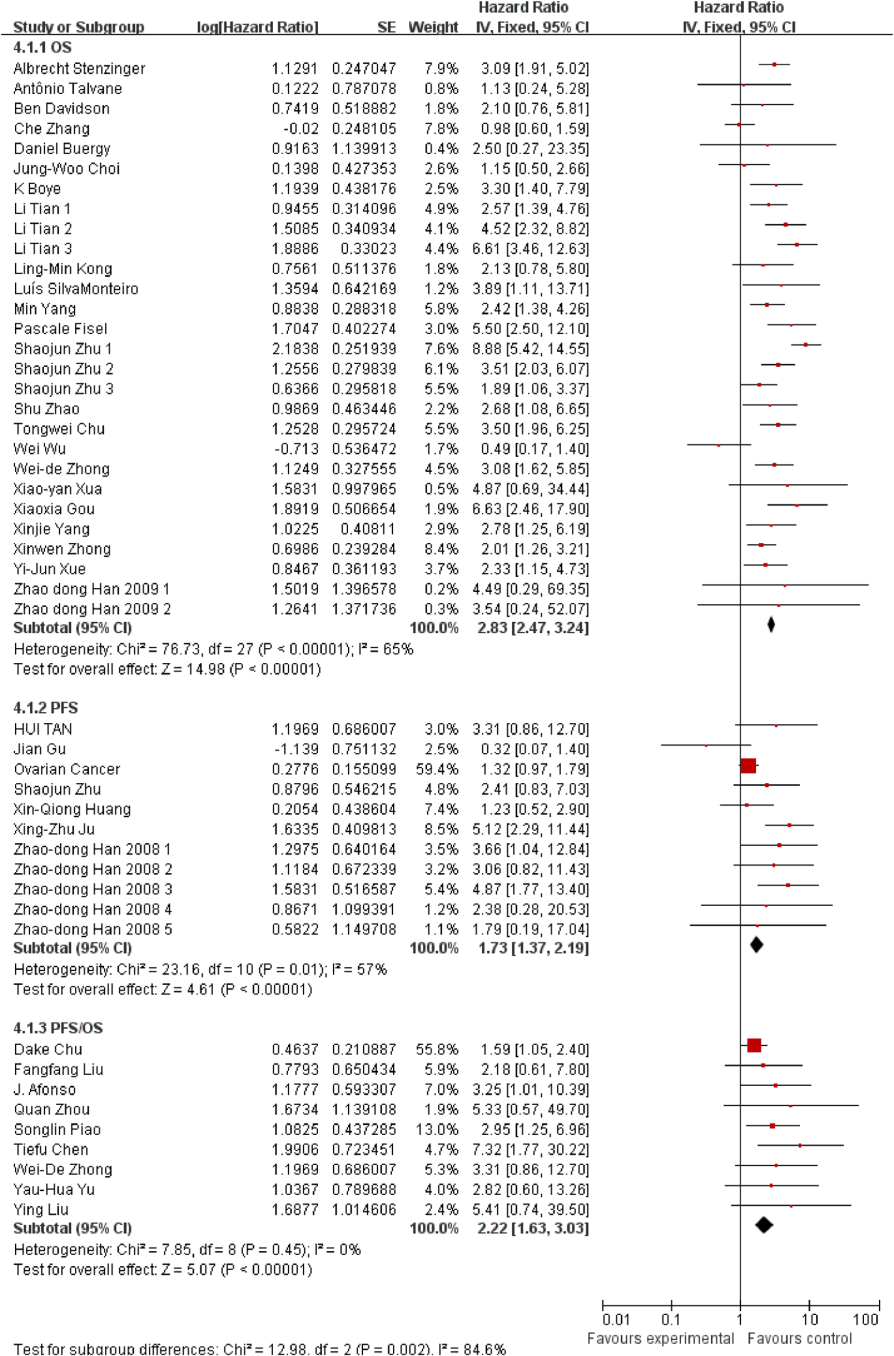
Subgroup analysis results based on survival condition

Besides, we also conducted subgroup analysis stratified by cancer type (Figure 3), ethnicity (Figure 4) and survival condition (Figure 5). Based on the cancer type group of studies, the investigation indicated that high EMMPRIN expression was associated with poor survival in bladder cancer (HR=2.21, 95% CI: 1.44-3.41, P<0.0001), prostate cancer (HR=3.54, 95% CI: 2.10-5.97, P<0.0001), gastrointestinal cancer (HR=2.96, 95% CI: 2.40-3.65, P<0.0001), breast cancer (HR=2, 75, 95% CI: 1.37-5.50, P<0.0001), cervical cancer (HR=2.63, 95% CI: 1.46-4.37, P<0.0001), hepatocellular cancer (HR=2.26, 95% CI: 1.09-4.69, P<0.0001), ovarian cancer (HR=1.37, 95% CI:1.02-1.83, P<0.0001), glioma (HR=2.77, 95%CI: 1.44-5.31, P=0.002) and others (HR=2.72, 95% CI: 1.88-3.95, P<0.0001). As for the population group of studies, both the Asian ethnicity (HR=2.63, 95% CI:2.32-2.99, P<0.0001) and Caucasian ethnicity (HR=2.04, 95% CI:1.65-2.63, P<0.0001), the EMMPRIN overexpression predicted a poor prognostic value in cancers. In addition, based on the survival condition, the subgroup results indicated that the high EMMPRIN was associated with OS (HR=2.83, 95% CI:2.47-3.24, P<0.0001), PFS (HR=1.73, 95% CI:1.37-2.19, P<0.0001) and OS/PFS (HR=2.22, 95% CI:1.63-3.03, P<0.0001). All the results summarized were presented in Table 2.

**Table 2:** Results of the overall and subgroup analyses for EMMPRIN overexpression and the outcome of cancer patients

Categories	No. of studies	Cases	Pooled HR	95% CI	P value
**Overall**	39	5739	2.46	2.21-2.75	<0.0001
**Cancer types**					
Bladder cancer	5	714	2.21	1.44-3.41	<0.0001
Prostate cancer	3	414	3.54	2.10-5.97	<0.0001
Gastrointestinal cancer	6	1459	2.96	2.40-3.65	<0.0001
Breast cancer	3	363	2.75	1.37-5.50	<0.0001
Cervical cancer	2	214	2.63	1.46-4.73	<0.0001
Hepatocellular cancer	2	104	2.26	1.09-4.69	<0.0001
Ovarian cancer	2	323	1.37	1.02-1.83	<0.0001
Others	18	2148	2.60	2.18-3.10	<0.0001
**Population**					
Asian	29	4382	2.63	2.32-2.99	<0.0001
Caucasian	10	1357	2.04	1.65-2.63	<0.0001
**Survival conditions**					
OS	23	3829	2.83	2.47-3.24	<0.0001
PFS	7	992	1.73	1.37-2.19	<0.0001
OS/PFS	9	918	2.22	1.63-3.03	<0.0001

Moreover, because the clinicopathological characteristics and driven factors are different in different cancers, we conducted a subgroup analysis during the tumor-stage-analysis based on cancer types (Supplementary Figure 1). The results indicated that high expression of EMMPRIN predicted an advanced tumor stage, which means our conclusion was relatively consistent, except for cervical cancer (HR=1.35, 95%CI: 0.73-2.48, p=0.33). According to our analysis, the expression of EMMRIN was not significantly associated with tumor stage in cervical cancer.

Besides, the cut-off value was not consistent among the studies included, thus we conducted a subgroup analysis based on the criteria of positive expression definition. The high cut-off value was identified when the percentage of positive cells is more than 50% or the scores are more than 3. And the low cut-off value was indentified when the percentage of positive cells is less than 50% and the scores are less than 3. Besides, 5 studies [35, 45, 46, 52, 53] enrolled in our meta-analysis provided no information of the cu-off value. Thus, these 5 studies were not included in the present subgroup analysis based on the criteria of positive expression definition. The results indicated that the high or low cut-off value didn’t affect our conclusion obviously (High: HR=2.76, 95%CI: 2.62-2.90, Low: HR=2.38, 95%CI: 2.33-2.44). Both the high cut-off value group and the low cut-off value group suggested the corresponding overexrepssion of EMMRIN predicted a poor prognosis outcome in cancers (Supplementary Figure 2).

### EMMPRIN overexpression and clinicopathological features

All the results assessing the association between clinicopathological features and EMMPRIN expression were presented in Table 3.

**Table 3:** Results of clinicopathological factors related to EMMPRIN overexpression

Subgroup	No. of studies	Cases	Pooled OR	95% CI	P value
Tumor stage (T3+T4/T1+T2)	24	4769	1.87	1.64-2.12	<0.0001
Differentiation (poor/ well +moderate)	14	3476	1.09	1.60-2.23	<0.0001
Clinical stage (III+IV/I+II)	25	4734	1.96	1.69-2.27	<0.0001
Nodal metastasis (negative/ positive)	12	2010	2.37	1.93-2.90	<0.0001

**Figure 6:**
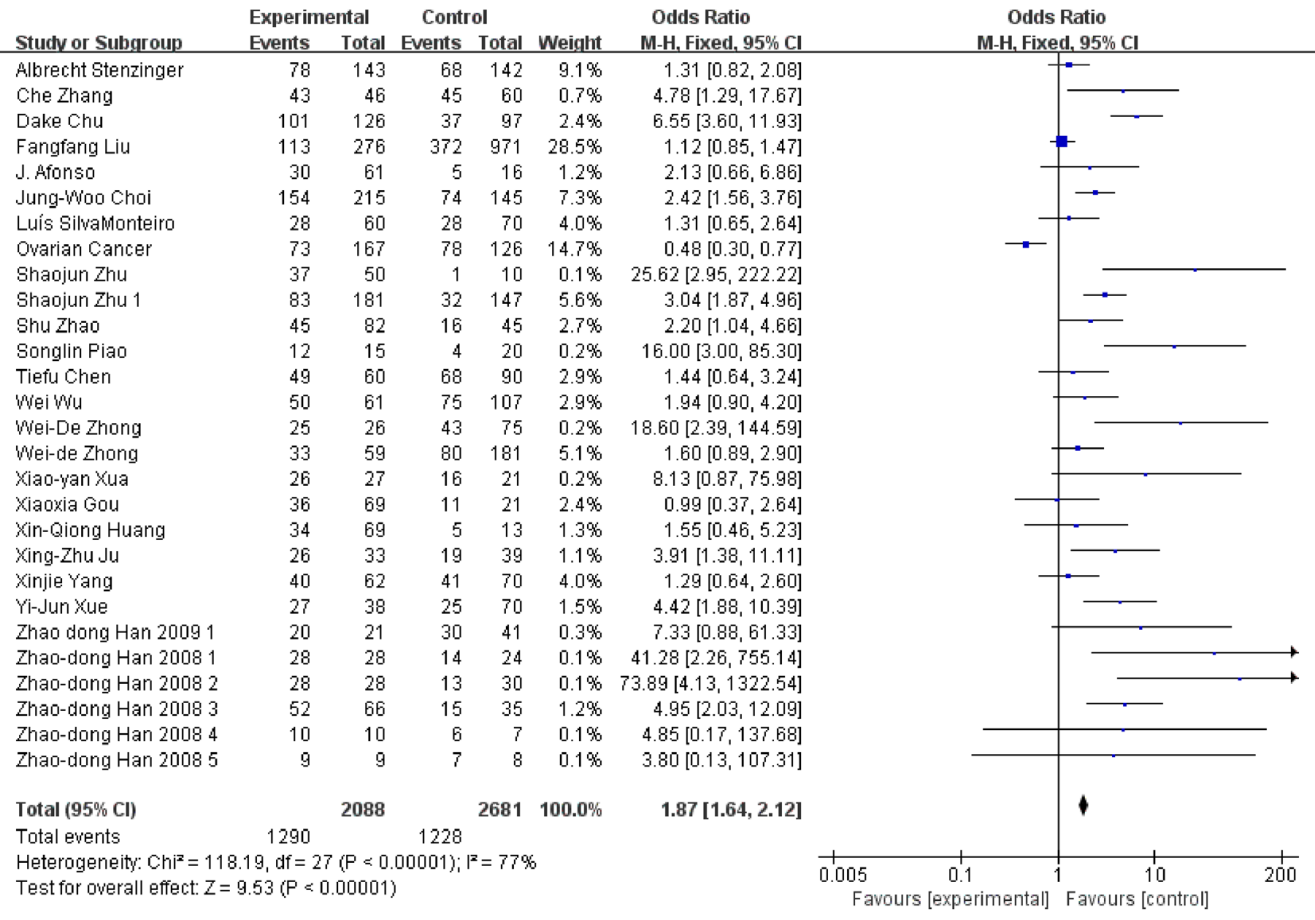
Association between EMMPRIN overexpression and tumor stage

**Figure 7:**
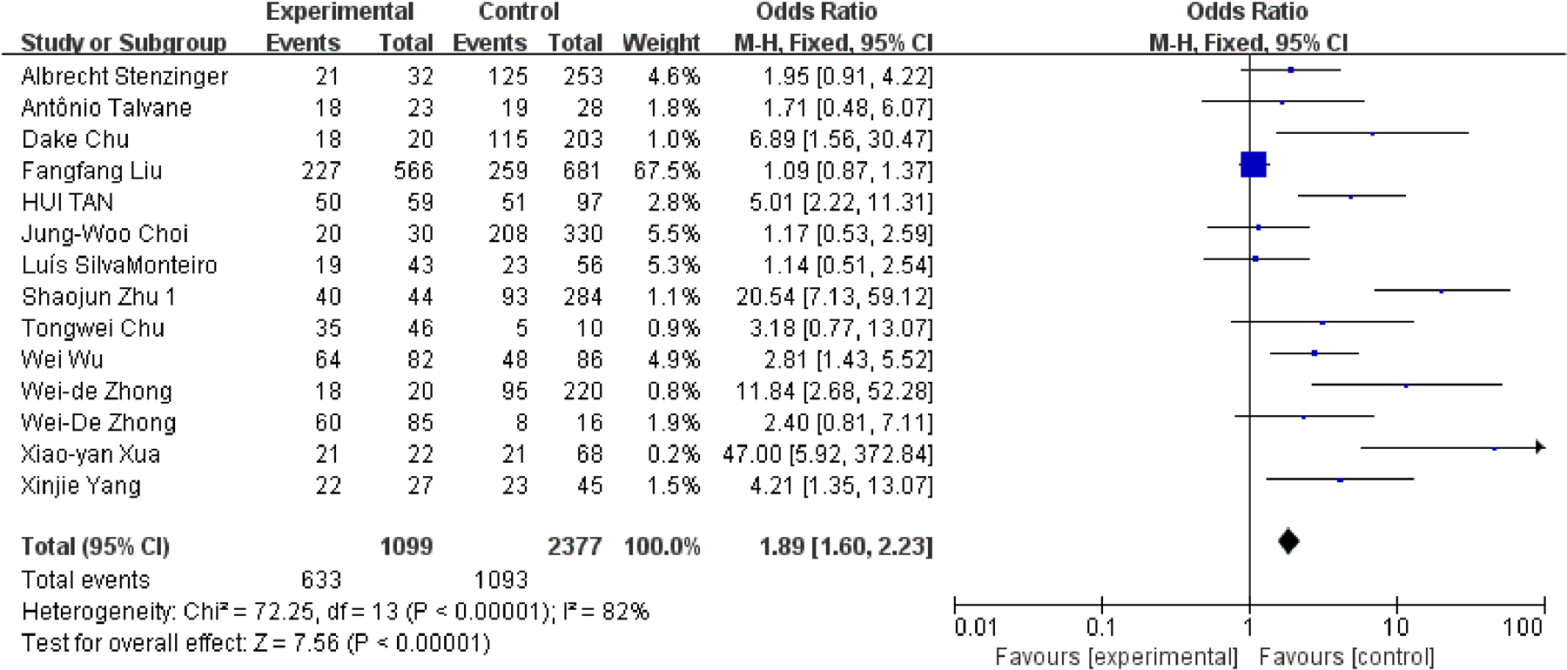
Association between EMMPRIN overexpression and tumor differentiation

**Figure 8:**
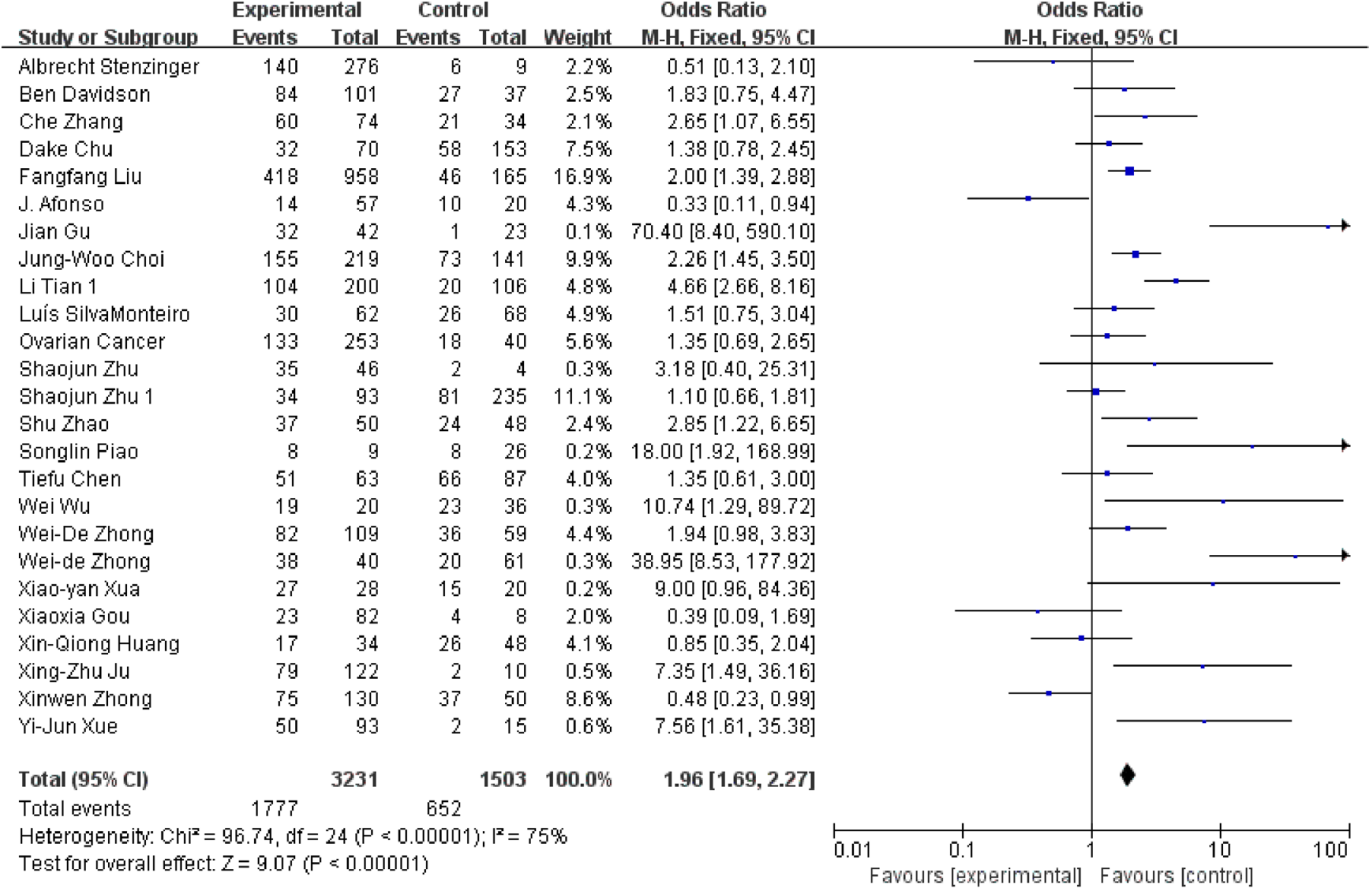
Association between EMMPRIN overexpression and clinical stage

**Figure 9:**
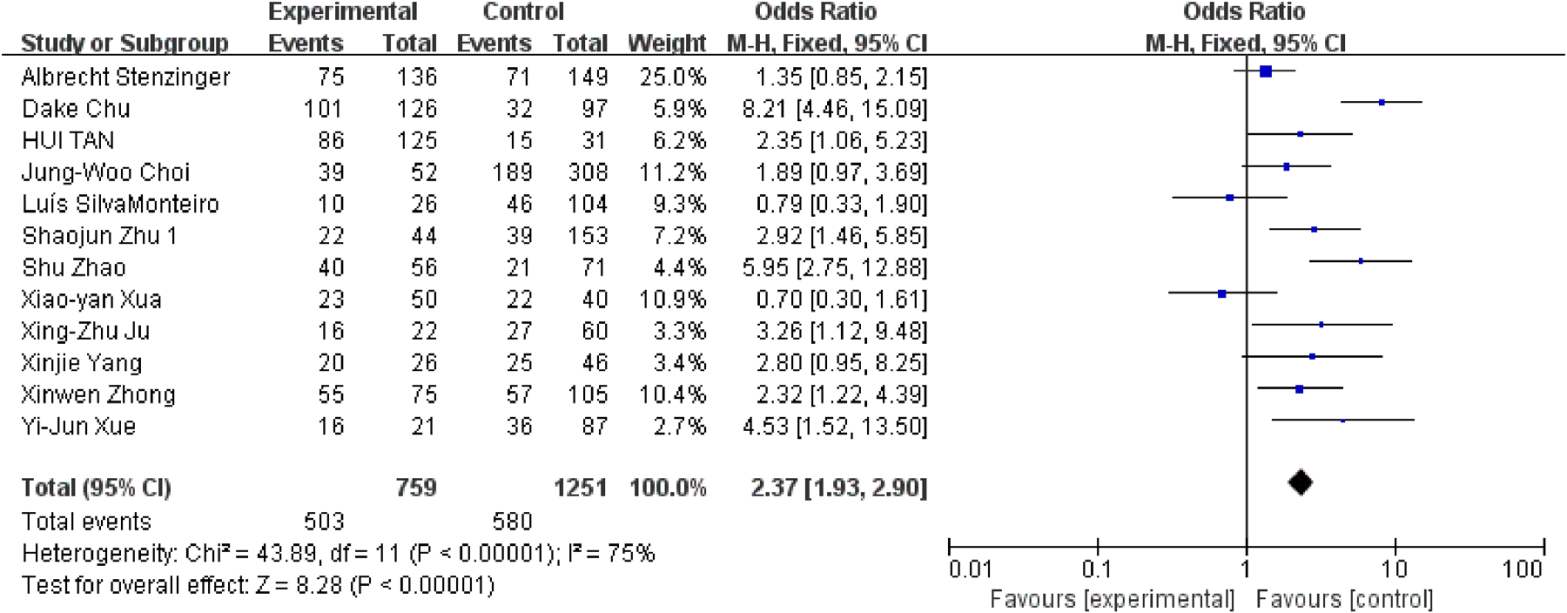
Association between EMMPRIN overexpression and nodal metastasis

We conducted analysis evaluating the clinicopathological features and EMMPRIN expression from the following aspects: tumor stage (Figure 6), differentiation (Figure 7), clinical stage (Figure 8) and nodal metastasis (Figure 9).

Among the included studies, 24 studies reported risk between high EMMPRIN expression and tumor stage. The results obviously indicated that the positive rate of EMMPRIN expression was significantly higher in cancers with tumor stage T3+T4 than with stageT1+T2 (OR=1.87, 95% CI:1.64-2.12, P<0.0001). Besides, the EMMPRIN overexpression was significantly associated with tumor differentiation (poor/ well+ moderate) (OR=1.09, 95% CI:1.60-2.23, P<0.0001). Stratified based on the clinical stage, the results showed a significant association between EMMPRIN expression and the risk of clinical stage III+IV than stage I +II (OR=1.96, 95% CI:1.69-2.27, P<0.0001). 12 studies compared the EMMPRIN expression negative nodal metastasis and positive nodal metastasis. The results showed that a higher EMMPRIN expression indicated a positive nodal metastasis (OR=2.37, 95% CI:1.93-2.90, P<0.0001).

### Quality assessment and sensitivity analysis

The quality of each study included in our meta-analysis was assessed using The Newcastle-Ottawa Scale (NOS). A star system was used to calculate the score of each study and a study award with 5 scores or more was considered as high quality article. The scores of the 39 studies include in our research ranged from 7 to 9.

**Figure 10:**
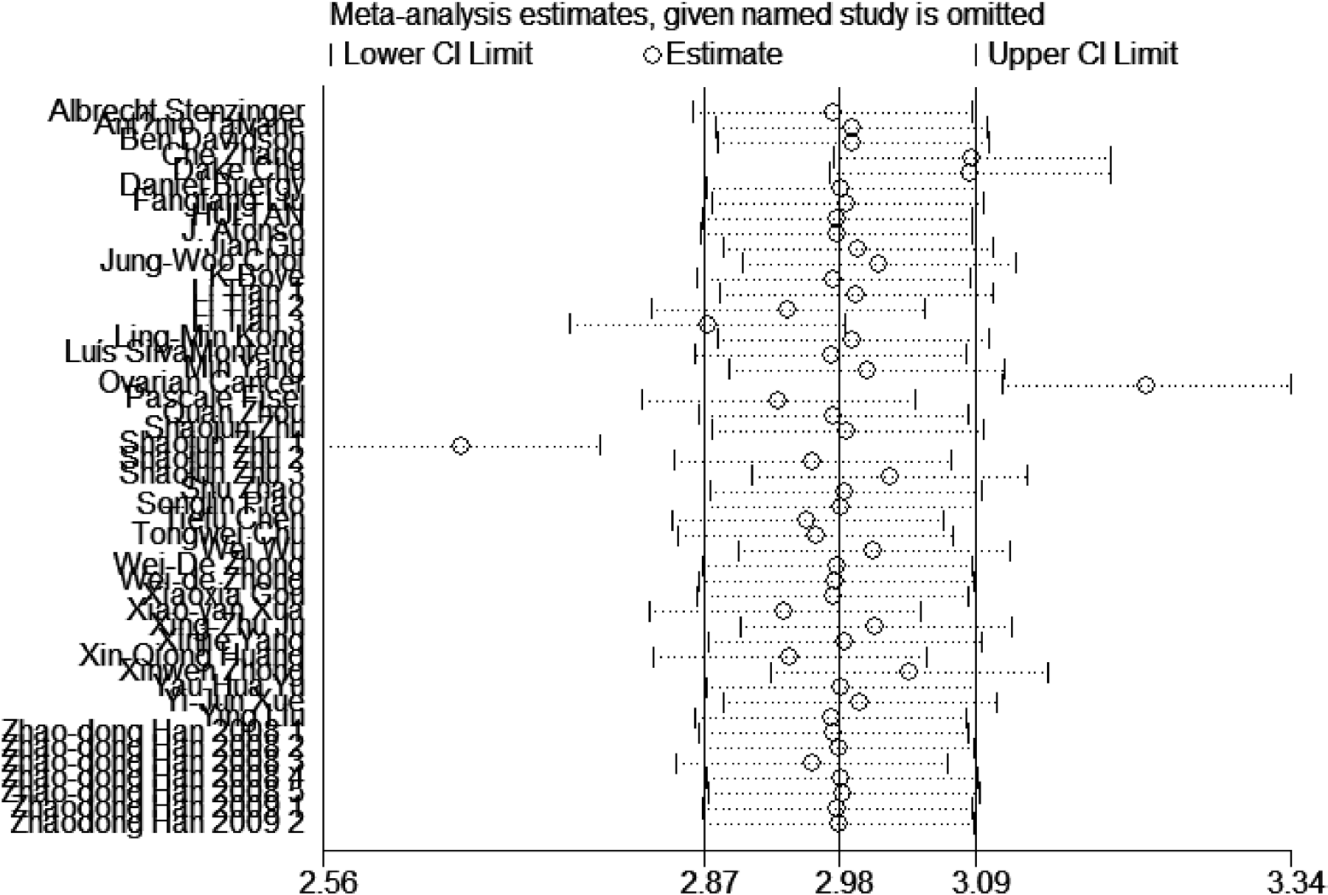
Sensitivity test among studies included

By omitting one individual study a time, sensitivity analysis between EMMPRIN overexpression and survival of cancer was conducted to investigate the potential sources of heterogeneity (Figure 10). The results indicated that overall risk estimate did not change, indicating a stable result of our meta-analysis.

### Publication bias

**Figure 11:**
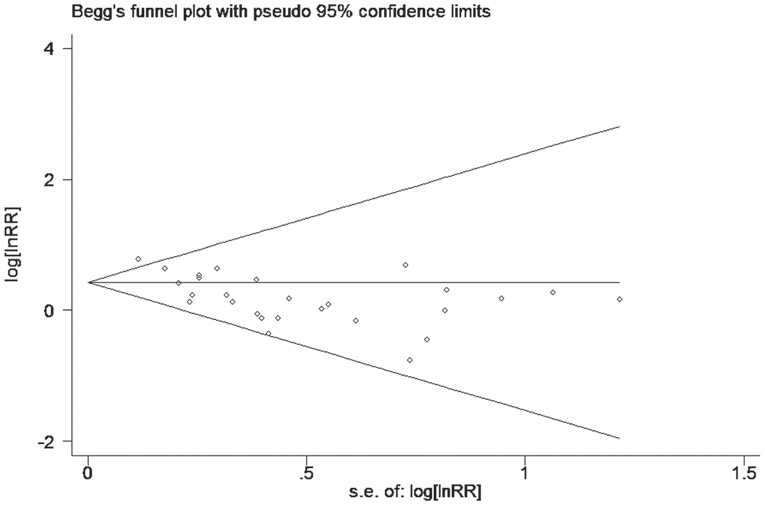
Funnel plot analysis investigating the publication bias between EMMPRIN overexpreession and cancer prognosis

According to the funnel plot (Figure 11), no evidence of obvious asymmetry existed. Furthermore, Begg’s funnel plot and Egger;s regression were also conducted to estimate the publication bias. The results (Table 4) showed no significant publication bias for pooled HR estimation. Similarly, there is no publication bias existed in the OR estimation and the subgroup of the analysis.

**Table 4:** Results of Egger’s and Begg’s tests

Comparison	N	Egger's test			Begg's test	
		t	*P*-value	95% CI	Z	*P*-value
Overall	48	1.4	0.167	(-0.30-1.69)	0.02	0.986
OS	28	-0.15	0.879	(-1.89-1.63)	0.18	0.859
PFS	11	1.31	0.224	(-0.73-2.71)	0.62	0.533
OS/PFS	9	4.67	0.002	(0.77-2.34)	1.15	0.251
Caucasian	10	1.71	0.127	(-0.47-3.12)	0.89	0.371
Asian	38	0.45	0.653	(-0.97-1.53)	0.62	0.538

## DISCUSSION

Most cancer deaths are due to metastasis with proliferation and angiogenesis [54]. Matrix metalloproteinases (MMPs), found in extracellular milieu of various tissues, are reported to be associated with poor survival of cancer patients [55]. Because of the specific structure, MMPs are responsible for the cancer metastasis, invasion, angiogenesis and tumorigenesis [56, 57]. And the MMPs are obviously up-regulated by the stimulated EMMPRIN, which makes EMMPRIN get involved with tumor metastasis [58]. It’s reported that EMMPRIN and MMP-9 can be found in normal keratinocytes [59] and tumor cells [60] and the expression of EMMPRIN is much higher in tumor tissues than the adjunct normal tissues [61]. Besides, EMMPRIN can interact with a verity of proteins, such as VEGF [62], lewis y antigen [63], caveolin-1 [64], cyclooxygenase-2 [65] and fascin [66], executing its effect on tumorigenesis by regulating tumor cell invasion, metastasis and adhesion.. Emerging evidence indicate that EMMPRIN is associated with prognosis of various cancers, however, the exact effects remains vaguely.

In the present study, the data from 39 studies with 5739 cases were analyzed to assess the association between EMMPRIN overexpression and its prognostic value in cancer. According to our analysis, EMMPRIN was significantly associated with poor outcome of cancer patients (HR=2.46, 95% CI: 2.21-2.75, P<0.0001). It’s been reported that in hepatocellular carcinomas, higher EMMPRIN expression correlates significantly with poor survival of patients. In breast cancer, the OS of patients with higher EMMPRIN expression was much shorter than those with lower EMMPRIN expression. The same situation also exists in other cancers. Our finding is consistent with the previous studies investigating the roles of EMMPRIN overexpression. Besides, our results revealed that higher expression of EMMPRIN was also an independent risk factor for the survival of cancer patients in Asian and Caucasian based on the subgroup stratified by ethnicity. The similar results are summarized when stratified by survival conditions.

To further investigate the prognostic value of EMMPRIN, the relationship between EMMPRIN expression and the clinicopathological factors was also analyzed in our meta-analysis. Our results suggested that higher EMMPRIN expression was obviously associated with worse clinicopathological features, including tumor stage (T3+T4/T1+T2), except for cervical cancer (HR=1.35, 95%CI:0.73-2.48, p=0.33), poor/ well+ moderate differentiation rate, clinical stage (III+IV / I +II) and nodal metastasis (negative/positive). This may also verify the strong association between EMMPRIN expression and the survival of tumor patients. This might be the first study to evaluate the clinicopathological significance of EMMPRIN in cancers. However the other clinicopathological factors, such as age, tumor location and sex, were not included in our analysis. Considering the complicacy of clinicopathological features, more studies on large populations are encouraged.

Because of the inconsistent method to test the EMMPRIN expression and positive criteria, we also analyzed the corresponding heterogeneity. Among all the 39 studies, 4 studies used TMA to detect the expression of EMMPRIN; the rest 35 studies used IHC to detect the expression of EMMPRIN, as indicated in our revised Table 1. Among these, 5 studies didn’t mention the cut-off value of positive expression of EMMPRIN. Both the percentage of positive cells and the intensity of staining scores were used according to different studies. However, the results indicated that our conclusion was relatively consistent. No obvious discrepancy was found during the analysis. Although the pathogeneses of different cancer types are divergent, our results could prove the prognostic value of EMMPRIN in cancers for the reasons below. First, high expression of EMMPRIN predicted worse overall survival in each sub grouped cancer. Second, elevated EMMPRIN expression was significantly associated with poor survival of cancer patients in a pooled analysis in all included cancers. It means that EMPPRIN might be a universally applicable biomarker in cancers.

The mechanism lied behind this correlation still remain unknown. MMPs stimulated by EMMPRIN in human cancers may account for one of these mechanisms. By activating signal transduction cascades through degrading extracellular matrix proteins, MMPs can enhance tumor metastasis and invasion [37]. It’s also been demonstrated that tumor progression could be inhibited by silencing EMMPRIN by RNA interference approach [67, 68]. In order to select a therapeutic strategy and to allocate medical resources with reasonableness, an accurate method to predict the prognosis of cancer patients is required [69]. Our meta-analysis concluded that EMMPRIN could be a prognostic marker in solid tumors.

However, some limitations still exist in result of the current meta-analysis. First, for subgroup analysis stratified by cancer type, some types have insufficient studies to summarize the main effect, such as gallbladder carcinoma and penis carcinoma. Second, several studies included used Engage Digitizer 4.1 to estimate the data because only Kaplan-Meier curve was provided, thereby leading to unavoidable calculation errors. Third, some clinicopathological factors, such as age, tumor location and sex, were not included in our analysis due to the insufficient data. Fourth, the cut-off values were inconsistent in the studies included, and this could be one source of heterogeneity. Therefore, more well-designed studies are needed to validate the findings of the current study.

In conclusion, EMMPRIN overexpression predicts a poor prognosis outcome of cancer patients and is significantly relevant to clinicopathological features. Therefore, EMMPRIN might be a reasonable prognostic bio-maker and therapeutic target of cancer.

## MATERIALS AND METHODS

There is no review protocol exists.

### Literature search

We comprehensively searched for published literature by consulting the electronic database PubMed, Cochrane Library databases and Web of Science before October 10, 2016, without language and publication restrictions. Studies were selected using the following terms: “Extracellular matrix metalloproteinase inducer” or “EMMPRIN” or “CD147” or “HAb18G” and “basigin” in combination with “cancer,” “tumor,” “carcinoma” and “neoplasm”. The references of retrieved articles were also reviewed for any potential eligible data and authors were contacted for specific information if necessary. Oncomine (User ID: 1610636@tongji.edu.cn) and TCGA (analyzed by cBioPortal) were searched to make our research complete. The literature search was performed independently by H. Fan and W. Yi with double check and consensus to resolve all the disagreements.

### Study selection

The studies were included if they met the following criteria: 1) the article enrolled should be case-control and cohort study; 2) expression of EPPRIN needs to be identified as positive with specific methods in cancer patients; 3) the relationship between EPPRIN expression and the time-to-event outcome, which was precisely defined, was reported; 4) sufficient data was provided to calculate the odds ratio (OR) and the hazard ratio (HR) with the corresponding 95% confidence intervals (CI) (ether directly obtained or indirectly calculated from Kaplan-Meier survival curves). Studies were ineligible if they were case reports, reviews, letters, duplicate studies, and articles without sufficient data. If more than one article focused on the same population, we preferred the latest one.

### Data extraction

Information was carefully extracted from all the eligible studies by two investigators (H. Fan and C. Wang) independently, including: the first author’s name, publication year, the ethnicity, cancer type, sample size, testing method, survival condition, duration of follow-up, EPPRIN expression data and the HRs and ORs with the corresponding 95% CI. Software Engauge Digitizer 4.1 was used to extract data if the study provided a Kaplan-Meier curve only.

### Quality assessment

We used Newcastle-Ottawa Quality Assessment Scale (NOS) to evaluate the quality of every study enrolled. Each item could be awarded with one point when meeting the requirement (total score ranged from 0 to 9). Studies got a score of 6 or more were considered to be of high quality.

### Statistical analysis

Review Manager 5.3 was used to perform all statistical analyses. Hazard ratios (HRs) and corresponding 95% confidence intervals (CIs) were used to evaluate the significance of the association between EPPRIN expression and the outcome of patients. The odds ratios (ORs) and corresponding 95%CI were used to analyze the correlation between EPPRIN overexpression and clinicopathological parameters, such as tumor stage, nodal metastasis and clinical grade. *Q*-test and *I*^*2*^ index were used to assess the heterogeneity between studies. A random-effects model was conducted when the heterogeneity was considered statistically significant (*P*<0.01). Otherwise, a fixed-effects model was conducted. Begg’s and Egger’s asymmetry tests were used to assess the potential publication bias. By omitting a study one time, sensitivity analysis was conducted to assess the stability of our results. Begg’s and Egger’s asymmetry tests and sensitivity analysis were performed by STATA software version 12.0 (STATA Corporation, College Station, TX, USA).

## SUPPLEMENTARY MATERIALS FIGURES



## Abbreviations

EMMPRIN: extracellular matrix metalloproteinase inducer
HR: hazard ratio
OR: odds ratio
MMPs: matrix metalloproteinases
VEGF: vascular endothelial growth factor
IHC: immunohistochemistry
CI: confidence interval
OS: overall survival
PFS: progression-free survival
