# Supplementary material for: The clinicopathological significance and prognostic value of EMMPRIN overexpression in cancers: evidence from 39 cohort studies
Source: Oncotarget. 2017 Jul 31;8(47):82643–60. doi: 10.18632/oncotarget.19740 (PMC5669917; doi:10.18632/oncotarget.19740)
Supplement: Supplementary file 1 [file oncotarget-08-82643-s001.pdf]

The clinicopathological significance and prognostic value of EMMPRIN overexpression in cancers: evidence from 39 cohort studies

SUPPLEMENTARY MATERIALS

Study or Subgroup	Experimental Events	Experimental Total	Control Events	Control Total	Weight	Odds Ratio M-H, Fixed, 95% CI
**6.1.1 bladder cancer**						
J. Afonso	30	61	5	16	1.2%	2.13 [0.66, 6.86]
Jung-Woo Choi	28	28	14	24	0.1%	41.28 [2.26, 755.14]
Wei-De Zhong	154	215	74	145	7.4%	2.42 [1.56, 3.76]
Xinjie Yang	25	26	43	75	0.3%	18.60 [2.39, 144.59]
Yi-Jun Xue	27	38	25	70	1.5%	4.42 [1.88, 10.39]
Zhao-dong Han 2008 1	0	0	0	0		Not estimable
**Subtotal (95% CI)**		**368**		**330**	**10.5%**	**3.36 [2.37, 4.77]**
Total events	264		161			
Heterogeneity: Chi² = 8.64, df = 4 (P = 0.07); I² = 54%						
Test for overall effect: Z = 6.80 (P < 0.00001)						
**6.1.2 prostate carcinoma**						
Wei-De Zhong	52	66	15	35	1.2%	4.95 [2.03, 12.09]
Zhao dong Han 2009 1	33	59	80	181	5.1%	1.60 [0.89, 2.90]
Zhao-dong Han 2008 3	20	21	30	41	0.3%	7.33 [0.88, 61.33]
**Subtotal (95% CI)**		**146**		**257**	**6.7%**	**2.47 [1.54, 3.95]**
Total events	105		125			
Heterogeneity: Chi² = 5.40, df = 2 (P = 0.07); I² = 63%						
Test for overall effect: Z = 3.77 (P = 0.0002)						
**6.1.3 colorectal cancer**						
Albrecht Stenzinger	78	143	68	142	9.2%	1.31 [0.82, 2.08]
Shaojun Zhu 1	83	181	32	147	5.7%	3.04 [1.87, 4.96]
**Subtotal (95% CI)**		**324**		**289**	**14.8%**	**1.97 [1.41, 2.74]**
Total events	161		100			
Heterogeneity: Chi² = 6.04, df = 1 (P = 0.01); I² = 83%						
Test for overall effect: Z = 3.99 (P < 0.0001)						
**6.1.4 cervical cancer**						
Xin-Qiong Huang	34	69	5	13	1.3%	1.55 [0.46, 5.23]
Xing-Zhu Ju	40	62	41	70	4.0%	1.29 [0.64, 2.60]
**Subtotal (95% CI)**		**131**		**83**	**5.3%**	**1.35 [0.73, 2.48]**
Total events	74		46			
Heterogeneity: Chi² = 0.07, df = 1 (P = 0.79); I² = 0%						
Test for overall effect: Z = 0.97 (P = 0.33)						
**6.1.5 others**						
Che Zhang	43	46	45	60	0.8%	4.78 [1.29, 17.67]
Dake Chu	101	126	37	97	2.5%	6.55 [3.60, 11.93]
Fangfang Liu	113	276	372	971	28.8%	1.12 [0.85, 1.47]
Luís SilvaMonteiro	28	60	28	70	4.1%	1.31 [0.65, 2.64]
Ovarian Cancer	73	167	78	126	14.8%	0.48 [0.30, 0.77]
Shaojun Zhu	37	50	1	10	0.1%	25.62 [2.95, 222.22]
Shu Zhao	45	82	16	45	2.8%	2.20 [1.04, 4.66]
Songlin Piao	12	15	4	20	0.2%	16.00 [3.00, 85.30]
Tiefu Chen	49	60	68	90	3.0%	1.44 [0.64, 3.24]
Wei Wu	50	61	75	107	2.9%	1.94 [0.90, 4.20]
Xiao-yan Xua	26	27	16	21	0.2%	8.13 [0.87, 75.98]
Xiaoxia Gou	36	69	11	21	2.4%	0.99 [0.37, 2.64]
Zhao-dong Han 2008 2	28	28	13	30	0.1%	73.89 [4.13, 1322.54]
Zhao-dong Han 2008 4	10	10	6	7	0.1%	4.85 [0.17, 137.68]
Zhao-dong Han 2008 5	9	9	7	8	0.1%	3.80 [0.13, 107.31]
**Subtotal (95% CI)**		**1086**		**1683**	**62.7%**	**1.54 [1.30, 1.83]**
Total events	660		777			
Heterogeneity: Chi² = 80.26, df = 14 (P < 0.00001); I² = 83%						
Test for overall effect: Z = 4.96 (P < 0.00001)						
**Total (95% CI)**		**2055**		**2642**	**100.0%**	**1.85 [1.62, 2.10]**
Total events	1264		1209			
Heterogeneity: Chi² = 115.92, df = 26 (P < 0.00001); I² = 78%						
Test for overall effect: Z = 9.27 (P < 0.00001)						
Test for subgroup differences: Chi² = 18.25, df = 4 (P = 0.001), I² = 78.1%						

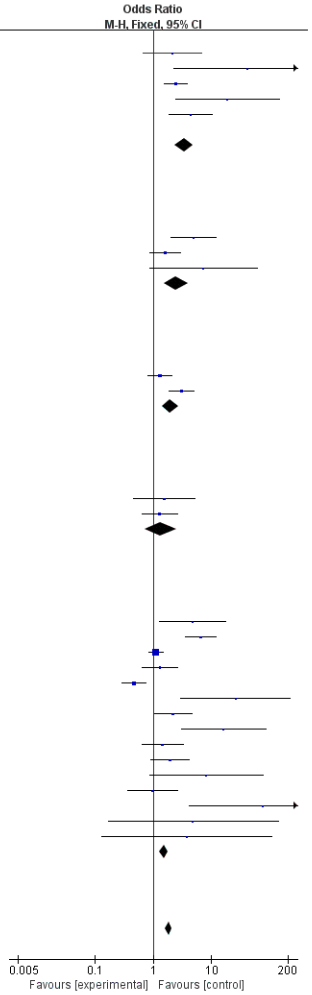

Supplementary Figure 1: Subgroup analysis of tumor stage based on cancer types.

Study or Subgroup	log[Hazard Ratio]	SE	Weight	Hazard Ratio IV, Fixed, 95% CI
**7.1.1 high cut-off value**				
Albrecht Stenzinger	0.122218	1.42712	0.0%	1.13 [0.07, 18.53]
Che Zhang	-0.0202	0.27444	0.1%	0.98 [0.57, 1.68]
Dake Chu	0.463734	0.04879	4.6%	1.59 [1.44, 1.75]
Daniel Buergy	0.916291	1.30933	0.0%	2.50 [0.19, 32.54]
HUI TAN	1.196948	0.065788	2.5%	3.31 [2.91, 3.77]
Jian Gu	-1.13943	2.20727	0.0%	0.32 [0.00, 24.21]
Jung-Woo Choi	0.139762	0.69315	0.0%	1.15 [0.30, 4.47]
Li Tian 1	0.945461	0.34359	0.1%	2.57 [1.31, 5.05]
Li Tian 2	1.508512	1.05779	0.0%	4.52 [0.57, 35.94]
Li Tian 3	1.888584	1.286474	0.0%	6.61 [0.53, 82.27]
Ling-Min Kong	0.756122	0.24846	0.2%	2.13 [1.31, 3.47]
Luís SilvaMonteiro	1.359437	0.10075	1.1%	3.89 [3.20, 4.74]
Min Yang	0.883768	0.300105	0.1%	2.42 [1.34, 4.36]
Pascale Fisel	1.704748	0.916291	0.0%	5.50 [0.91, 33.14]
Quan Zhou	1.673351	0.56212	0.0%	5.33 [1.77, 16.04]
Shaojun Zhu	0.879627	0.476234	0.0%	2.41 [0.95, 6.13]
Songlin Piao	1.082483	0.223144	0.2%	2.95 [1.91, 4.57]
Wei Wu	-0.71335	1.56065	0.0%	0.49 [0.02, 10.44]
Xiao-yan Xua	1.583094	0.75502	0.0%	4.87 [1.11, 21.39]
Xiaoxia Gou	1.891906	0.898941	0.0%	6.63 [1.14, 38.62]
Xing-Zhu Ju	0.205387	0.65393	0.0%	1.23 [0.34, 4.42]
Xinwen Zhong	0.698632	0.229523	0.2%	2.01 [1.28, 3.15]
Yi-Jun Xue	0.846726	0.138892	0.6%	2.33 [1.78, 3.06]
Zhao-dong Han 2008 1	1.297463	0.039221	7.1%	3.66 [3.39, 3.95]
Zhao-dong Han 2008 2	1.118415	0.19845	0.3%	3.06 [2.07, 4.51]
Zhao-dong Han 2008 3	1.583094	0.57098	0.0%	4.87 [1.59, 14.91]
Zhao-dong Han 2008 4	0.8671	1.07881	0.0%	2.38 [0.29, 19.72]
Zhao-dong Han 2008 5	0.582216	1.51413	0.0%	1.79 [0.09, 34.81]
**Subtotal (95% CI)**			**17.4%**	**2.76 [2.62, 2.90]**
Heterogeneity: Chi² = 229.15, df = 27 (P < 0.00001); I² = 88%				
Test for overall effect: Z = 40.43 (P < 0.00001)				
**7.1.2 low cut-off value**				
Fangfang Liu	0.779325	0.4943	0.0%	2.18 [0.83, 5.74]
J. Afonso	1.177731	0.014889	49.3%	3.25 [3.15, 3.34]
K Boye	1.193922	0.336472	0.1%	3.30 [1.71, 6.38]
Ovarian Cancer	0.277632	0.0202	26.8%	1.32 [1.27, 1.37]
Shaojun Zhu 1	2.183802	1.708378	0.0%	8.88 [0.31, 252.69]
Shaojun Zhu 2	1.255616	0.708036	0.0%	3.51 [0.88, 14.06]
Shaojun Zhu 3	0.636577	0.058269	3.2%	1.89 [1.69, 2.12]
Tiefu Chen	1.99061	0.173953	0.4%	7.32 [5.21, 10.29]
Tongwei Chu	1.252763	0.470004	0.0%	3.50 [1.39, 8.79]
Wei-de Zhong	1.196948	0.065788	2.5%	3.31 [2.91, 3.77]
Xin-Qiong Huang	1.022451	0.223144	0.2%	2.78 [1.80, 4.31]
Xinjie Yang	1.633545	0.941178	0.0%	5.12 [0.81, 32.40]
Yau-Hua Yu	1.036737	0.51083	0.0%	2.82 [1.04, 7.67]
Zhao dong Han 2009 1	1.501853	1.23787	0.0%	4.49 [0.40, 50.81]
Zhao dong Han 2009 2	1.264127	1.42712	0.0%	3.54 [0.22, 58.05]
**Subtotal (95% CI)**			**82.6%**	**2.38 [2.33, 2.44]**
Heterogeneity: Chi² = 1373.46, df = 14 (P < 0.00001); I² = 99%				
Test for overall effect: Z = 75.57 (P < 0.00001)				
**Total (95% CI)**			**100.0%**	**2.44 [2.40, 2.50]**
Heterogeneity: Chi² = 1630.37, df = 42 (P < 0.00001); I² = 97%				
Test for overall effect: Z = 85.54 (P < 0.00001)				
Test for subgroup differences: Chi² = 27.76, df = 1 (P < 0.00001), I² = 96.4%				

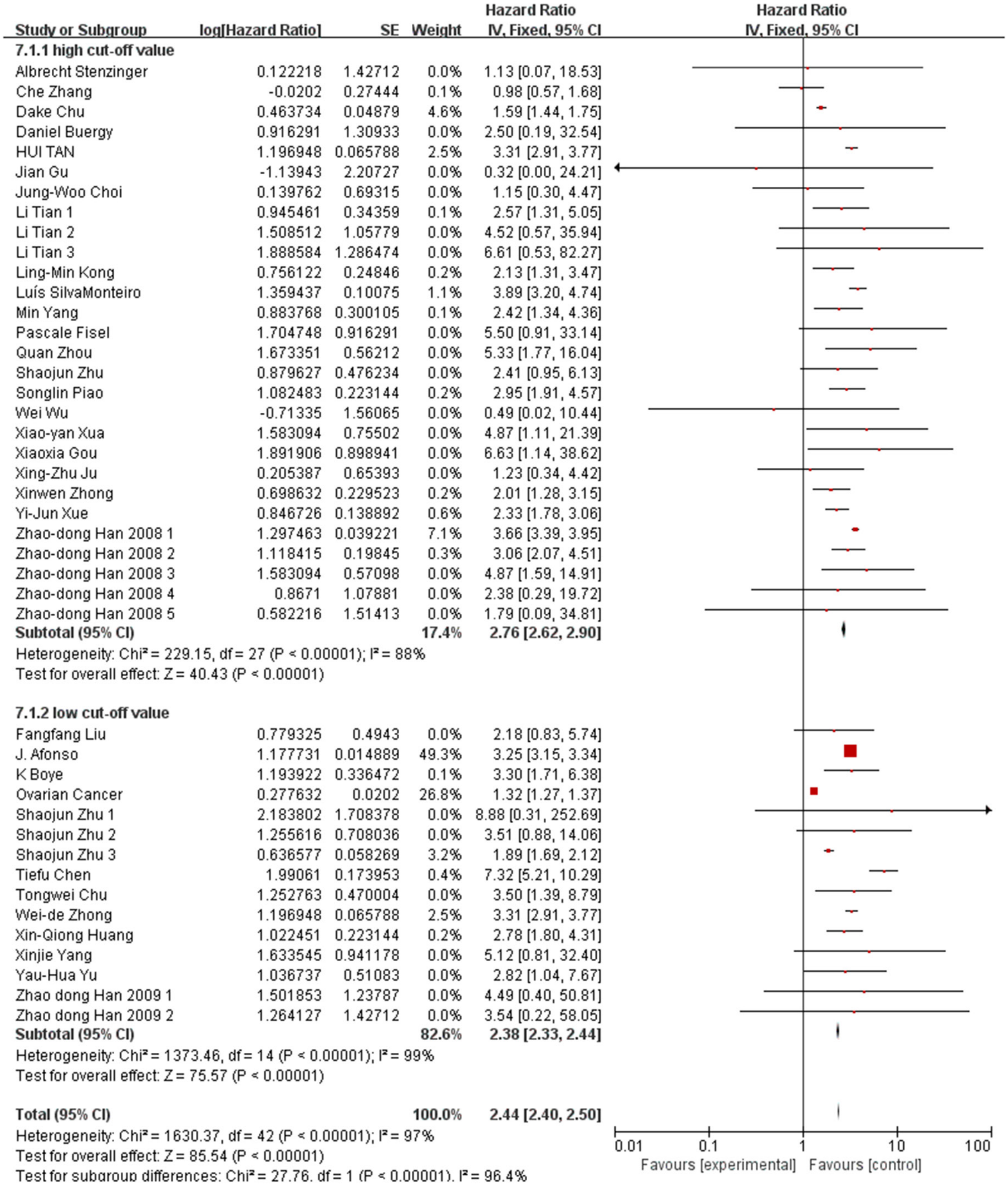

Supplementary Figure 2: Subgroup analysis based on the EMMPRIN positive expression determination.
